# Association between urinary iodine excretion, genetic disposition and fluid intelligence in children, adolescents and young adults: the DONALD study

**DOI:** 10.1007/s00394-023-03152-6

**Published:** 2023-04-27

**Authors:** Christina-Alexandra Schulz, Leonie Weinhold, Matthias Schmid, Markus M. Nöthen, Ute Nöthlings

**Affiliations:** 1 Institute of Nutrition and Food Sciences, Nutritional Epidemiology, University of Bonn, Bonn, Germany; 2 Department of Medical Biometry, Informatics and Epidemiology, University Hospital Bonn, University of Bonn, Bonn, Germany; 3 Institute of Human Genetics, School of Medicine, University of Bonn, University Hospital Bonn, Bonn, Germany

**Keywords:** Cognition, Fluid intelligence, General cognitive function, Urinary iodine, Genetic disposition, Polygenic score

## Abstract

**Purpose:**

Iodine deficiency increases the risk of cognitive impairment and delayed physical development in children. It is also associated with cognitive impairment in adults. Cognitive abilities are among the most inheritable behavioural traits. However, little is known about the consequences of insufficient postnatal iodine intake and whether the individual genetic disposition modifies the association between iodine intake and fluid intelligence in children and young adults.

**Methods:**

The cultural fair intelligence test was used to assess fluid intelligence in the participants of the DONALD study (*n* = 238; mean age, 16.5 [SD = 7.7] years). Urinary iodine excretion, a surrogate iodine intake marker, was measured in 24-h urine. Individual genetic disposition (*n* = 162) was assessed using a polygenic score, associated with general cognitive function. Linear regression analyses were conducted to determine whether Urinary iodine excretion was associated with fluid intelligence and whether this association was modified by individual genetic disposition.

**Results:**

Urinary iodine excretion above the age-specific estimated average requirement was associated with a five-point higher fluid intelligence score than that below the estimated average requirement (*P* = 0.02). The polygenic score was positively associated with the fluid intelligence score (*β* = 2.3; *P* = 0.03). Participants with a higher polygenic score had a higher fluid intelligence score.

**Conclusion:**

Urinary iodine excretion above the estimated average requirement in childhood and adolescence is beneficial for fluid intelligence. In adults, fluid intelligence was positively associated with a polygenic score for general cognitive function. No evidence showed that the individual genetic disposition modifies the association between Urinary iodine excretion and fluid intelligence.

## Introduction

Sufficient availability of iodine is essential for the correct development and adequate functioning of the human brain [1]. Iodine deficiency, leading to inadequate thyroid hormone production, can be observed across the lifespan. Iodine deficiency during pregnancy and infancy may impair the growth and neurodevelopment of the offspring and increase infant mortality. In children and adolescents, iodine deficiency may increase the risk of impaired cognitive function and delayed physical development. In adults, iodine deficiency has been associated with cognitive impairment [2]. Moreover, in adults, secondary to hypothyroidism, moderate-to-severe iodine deficiency may cause subtle but widespread adverse effects, including decreased educability, apathy and reduced work productivity [1]. Thus, iodine deficiency may be considered one of the most significant single causes of preventable brain damage worldwide [1].

Genetic factors contribute significantly to human cognitive performance. Heritability estimates for cognitive function range from 12 and 25% in adults [3–5] and from 22 to 46% in children [6]. Today, more than 140 loci across the genome have been associated with general cognitive function in genome-wide association studies (GWAS) in adults [4] and children [7]. Strong evidence shows a substantial genetic influence on cognitive traits [3–5]; however, a few studies have shown that iodine deficiencies after birth are associated with cognitive performance in children [8, 9] and adolescents [10], and little is known about the possible interaction between genetic factors and iodine intake regarding cognitive performance. Therefore, we investigated whether urinary iodine excretion (UIE), as a surrogate marker of iodine intake, was associated with measures of fluid intelligence (FI), as a measure of executive cognitive functioning, in the participants of the Dortmund Nutritional and Anthropometric Longitudinally Designed (DONALD) study. More specifically, a polygenic score (PGS) was used to consider the individual genetic disposition of general cognitive function. Thus, we tested whether individual genetic disposition using the PGS [11] may modify the association between UIE and cognitive performance.

### Methods

#### Study design

The DONALD study is an ongoing cohort study that collects information on the diet, growth, development and metabolism of healthy children and adolescents since 1985 in Dortmund, Germany. Annually, 35–40 infants at the age of 3 months are newly recruited. Healthy German infants (i.e. infants free of diseases that affect growth and/or dietary intake) whose parents are willing to participate in a long-term study and of whom at least one has sufficient knowledge of the German language are eligible for inclusion in the study. Annual examinations include 3-day weighed dietary records, anthropometric measurements, collection of 24-h urine samples (starting at the age of 3–4 years) and interviews on lifestyle and medical examinations. Detailed information on the DONALD study can be found elsewhere [12, 13]. The DONALD is registered at the German Clinical Trials Registry (DRKS-ID DRKS00029092 https://drks.de/search/de/trial/DRKS00029092). The metadata for the DONALD study is available at the following repository https://mica.mdc-berlin.de/study/donald. In addition to regular examinations, add-on measurements (including quantification of urinary iodine excretion, assessment of cognitive function, or generation of genetic data) are performed in subsets of the study population.

The study was approved by the Ethics Committee of the University of Bonn and conducted according to the Declaration of Helsinki. All examinations were performed with parental and later on children’s written consent.

#### Study population

**Fig. 1:**
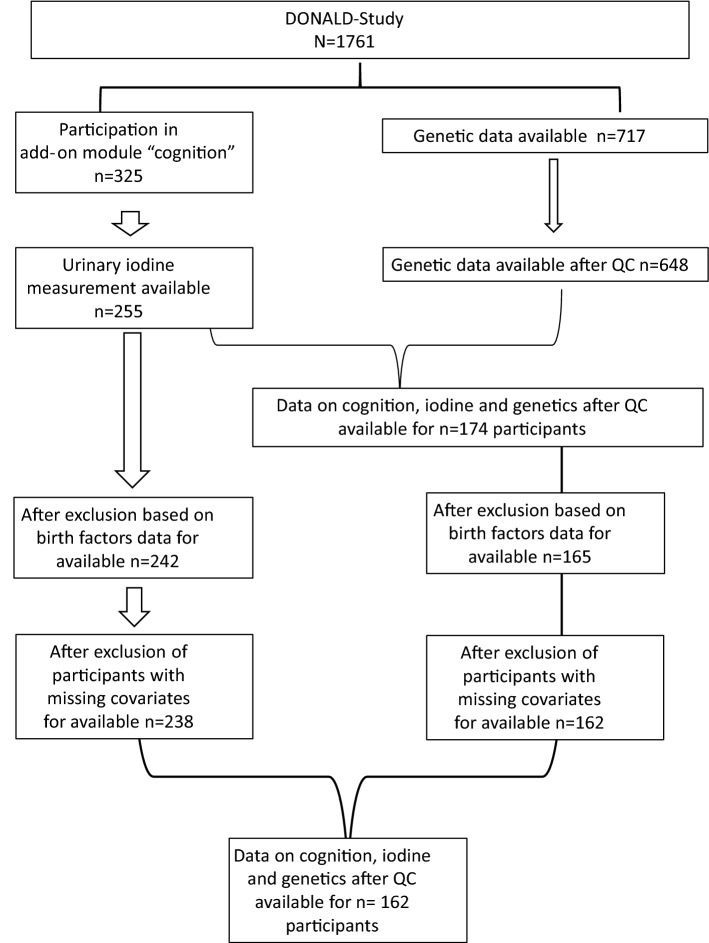
Flowchart of the DONALD participants included in the study

In 2017 and 2018, the add-on module ‘cognition’ was included in the DONALD study. Participants with data available from this module (*n* = 325) were included in the present study. Participants were excluded from the analysis if urine measurements were not available (*n* = 70) or if they were born preterm (≤ 36 weeks), with low birthweight < 2500 g, or as children of multiple births (*n* = 13), which resulted in 242 participants. Information on covariates (birthweight, pregnancy duration and exclusive breastfeeding) was missing in *n* = 4 participants. Hence, in total, 238 participants were included in the final analysis of the association between UIE and cognitive function. Genetic data were available for 717 DONALD study participants, of which 648 were included after quality control. Of these, 162 participants took part in the add-on module ‘cognition’, had their UIE measured, were born as single-term babies with a birthweight of > 2500 g and had no missing covariate values (Fig. 1).

#### Variable assessment

##### Fluid intelligence

In 2017 and 2018, as part of an add-on module, the German versions of the language-free and figure-based culture fair intelligence tests (CFTs) were used to assess FI [14, 15]. The CFT 1-R was used for participants aged < 8.5 years, whereas the CFT 20-R was used for participants aged ≥ 8.5 years. The tests had a multiple-choice format, and all items were sorted by increasing difficulty. FI scores were calculated based on the results from the age-specific CFTs.

### CFT 1-R

The interview-based paper-and-pencil test measured FI in three subtests (figural task continuation, classification and matrices) which included 15 tasks each. It was reported to take approximately 40 min to complete. Correct answers across all three subtests were summed up to a raw score. This was converted into an age-standardised FI score. The CFT 1-R was shown to have a high reliability (*r* = 0.97) [14].

### CFT 20-R

This self-administered computer test was reported to take approximately 1 h. It consists of two parts (56 and 45 items), including the following figural tasks: continuation of series, classifications, matrices and topological conclusions. Correct answers across both parts were summed up and converted into an age-standardised FI score. The reliability of the CFT 20-R was high (*r* = 0.95) [15].

### Urinary iodine excretion

In the DONALD study, urine collections are performed annually following a standardised procedure, which was previously described [16, 17], by the time children have learnt to use the toilet (mostly at the age of 3–4 years) [13]. The 24-h urine collection is described in detail elsewhere [18]. Briefly, the participants were instructed to void their bladder in the morning. This micturition is discarded, and the time is noted as the start time of the 24-h urine collection. For the next 24 h, all micturitions were collected, including the first void of the following morning. Samples were immediately stored in preservative-free, Extran-cleaned (Extran, MA03; Merck, Darmstadt, Germany) 1-L plastic containers at less than − 12 °C before transfer by a dietician to the DONALD study centre. At the institute, the urine samples were stored at − 20 °C until analysed [16]. After thawing, combining urine contents of > 1 containers and thorough mixing, the total urine volume was determined. From each 24-h urine sample, several aliquots of 20 mL were stored at − 22 °C for further analyses and as a reserve in the DONALD urine bank [19]. Information on storage stability was reported elsewhere [19]. Before pipetting, the sample was shortly stirred for the particular measurement [19]. After acidic wet washing of the samples, the urinary iodine (μg/L) concentration was determined by a modified Sandell–Kolthoff method [20]. The methods were validated against the gold standard analytics for iodine measurements, i.e. the inductively coupled plasma mass spectrometry (ICP-MS). In the most relevant concentration range of 50–100 µg/L, the Sandell–Kolthoff method was in close agreement with the ICP-MS. A previous investigation within the DONALD study showed that the intraclass correlation in comparison with ICP-MS was 0.91 [16, 21]. The DONALD laboratory has participated in the in validation checks of the EQUIP programme [16, 21].

To further minimise potential errors from urine collection, information on the actual total collection interval in minutes was determined. UIE was calculated by the following equation:

$${\text{UIE }}\left( {{{\mu g} \mathord{\left/ {\vphantom {{\mu g} d}} \right. \kern-0pt} d}} \right) \, = \;\frac{{\left( {\frac{{{\text{urine volume }}\left( L \right)\; * \;{\text{urinary ioidine}}\;\frac{\mu g}{{dl}} }}{100} } \right)}}{{\text{sampling time}}} * 1440.$$

Moreover, samples with daily creatinine excretion < 0.1 mmol/kg body weight were excluded from the analysis [16]. Based on their UIE, participants were categorised according to the estimated average requirement (EAR) of iodine intake [22]. Assuming 15% non-renal iodine losses, participants with a UIE < 55, 62, or 81 μg for participants aged < 8, 13 and 18 years, respectively, were categorised as having an iodine intake below the EAR.

For our main analysis, we considered the latest UIE before the FI measurement. For sensitivity analysis, we used pooled data from up to three urine samples before the FI measurement. In 32 participants, data for only two measurements were available. For each participant, the mean UIE was calculated by summing up the individual UIE levels divided by the number of samples available.

### Genetic data and construction of the PGS

Since 2015, saliva (participants aged < 18 years) is collected by oral swab, and blood samples is obtained by venipuncture (participants aged > 18 years) within the add-on module ‘Genetic’. Blood plasma was extracted by centrifugation, and samples were stored in factions of 500 μL at – 80 °C in the in-house biobank of the DONALD study centre in Dortmund. Likewise, saliva was aliquoted and frozen at − 80 °C. DNA from the lymphocytes and saliva was extracted at the Life & Brain GmbH (Bonn) according to the manufacturer’s instructions using a Chemagic Magnetic Separation Module I or by salting out (prepIT purifier von Oragene). Genotyping was performed using the Illumina Infinium Global Screening Array v2.0. At present, genetic data are available for 717 participants from the DONALD study. Data were imputed using the Haplotype Reference Consortium panel [23, 24] up to a total of 39,131,578 genetic markers. During quality control, all variants with an imputation quality of < 0.3 were removed (*n* = 7,640,630). In addition, single-nucleotide polymorphisms (SNPs) with missing genotype information > 0.05 (*n* = 713,941) were removed when deviating from Hardy–Weinberg equilibrium (*P* < 0.000001; *n* = 78) or if they were rare variants (minor allele frequency < 0.05; *n* = 4,658,147). This led to a total of 4,658,147 genetic variants in 648 participants, which were used to construct the PGS. The PGS was constructed by first clumping the SNPs to capture those that have the lowest p-values (based on the GWAS of Davies et al. [3]) within a linkage disequilibrium block (up to an *r*^2^ = 0.2 among the genetic variants, range 1000 kb). The PGS was then calculated by summing the effect size-weighted number of associated alleles for each of the 162 participants with available data on FI. The best-fit *P* value threshold for the PGS was defined by the highest *r*^2^ value of the crude FI–PGS association (here: 0.3). Finally, the PGS was z-standardised.

### Covariates

The time between urinary iodine measurement and FI assessment via the CFT was quantified in years. Birth characteristics, including pregnancy duration (weeks) and birthweight (g), were extracted from the ‘Mutterpass’, a standardised document given to all pregnant women in Germany. Breastfeeding practice and duration were assessed by the study paediatricians at each of the first visits at 3, 6, 9, 12 and 18 months until the infant was fully weaned. To distinguish between full and partial breastfeeding, consumption of any additional liquids, formula or food was inquired about. Full breastfeeding duration (weeks) was defined as the number of weeks of both exclusive (breast milk only with no other food or drink) and predominant (breast milk in combination with water or water-based drinks) breastfeeding. Participants were grouped into those with < 3 weeks (= 0) and ≥ 3 weeks (= 1) of full breastfeeding. In regular intervals, parents were interviewed about their socio-economic characteristics such as maternal and paternal education. Parents were classified as having a higher education (= 1) if they had attended school for ≥ 12 years; otherwise, they were classified as having no higher education (= 0).

### Power calculation

With the given sample sizes of 238 and 162 participants, respectively, considering a significance level of 0.05 and a power of 80%, the minimum detectable difference between participants with UIE ≥ EAR compared with participants with a UIE < EAR were 2 and 2.5 points, respectively, in the FI score. Power calculations were performed by simulations using linear regression analysis adjusted for the PGS (standard normally distributed) and additional eight covariates (four that were standard normally distributed each and four that were Bernoulli distributed with probability of 0.5 each).

### Statistics

The baseline characteristics of the study population are presented as means with standard deviations (SDs) for continuous variables and counts with percentages for categorical variables. Linear regression models were used to test the association between FI (i.e. FI score) and UIE (categorised in < EAR vs. ≥ EAR) as well as the PGS. First, univariable regression models were fitted to investigate the basic associations of FI with UIE and PGS. Second, the linear regression models were adjusted for age, birth factors (i.e. birthweight, pregnancy duration, and exclusive breastfeeding), time between the measurements, CFT version (CFT-1R vs. CFT-20-R) and parental higher education. Analyses including the PGS were adjusted for the first five principal components of ancestry. Then, whether the association between FI and UIE or the PGS changed substantially when mutually adjusting for UIE and PGS was examined. Finally, whether the association between the PGS and FI remained when it was adjusted for further confounders (i.e. birth factors, time between the measurements, CFT version and parental education) was tested. Whether an interaction between UIE and PGS existed was assessed by including a product term (UIE x PGS) in the regression models, adjusting for birth factors, time in-between the measurements, CFT version and parental education. Results of linear regression models are presented by beta coefficients (*β*), their confidence intervals (CI) and *p* values (*P*). The beta coefficient refers to the estimated average change in the outcome variable when the independent variable increases by one unit.

In the sensitivity analysis, the analyses were repeated using the mean UIE from up to three urine samples ahead of the FI measurement, as a proxy for habitual iodine exposure. In addition to the categorical UIE variable, we used age- and sex-standardised levels, i.e. *z*-score transformed, of UIE (zUIE) to compare the effect of 1 SD increase in either UIE or PGS.

*P* values were two-sided and considered statistically significant if ≤ 0.05. All analyses were performed using the R Software for Statistical Computing, version 4.1.2 [25].

## Results

The characteristics of the 242 participants are shown in Table 1. The study included more female (55%) than male participants. The mean (SD) age was 7.2 (0.8) and 19.0 (7.1) years when FI was assessed using the CFT-1 and CFT-20R, respectively. The overall mean FI score was 110 (14.0) points on the CFT scales. The mean UIE was 59 (27) μg/24 h and 100 (53) μg/24 h for the younger (CFT-1) and older (CFT-20R) participants, respectively. The majority (66%) of the participants had a UIE above the EAR, and this was the case for 41% and 72% of the younger (CFT-1) and older (CFT-20R) participants, respectively. The median time in-between the measurement of UIE and CFT administration was 2.8 [IQR: 0.6–4.5] years; however, it was lower (median 0.0 [IQR: 0.0–0.9] years) for the younger participants (CFT-1) than for the older (CFT-20R) ones (median 3.1 [1.4–5.3] years). Most of the participants (87%) were exclusively breastfed. On average, participants were exclusively breastfed for 19.0 (9.4) weeks, and the majority of the participants’ fathers had a high education level (73% and 76%). The education level of the majority of participants’ mothers was also high (98% and 71%).

**Table 1:** Characteristics^1^ of the participants from the DONALD study

	All (*n* = 242)	CFT-1 (*n* = 44)	CFT-20R (*n* = 198)	Missing
Male sex, *n* (%)	117 (48%)	24 (55%)	93 (47%)	0
Pregnancy duration (week)	40 (± 1.3)	39 (± 1.3)	40 (± 1.4)	0
Birthweight (g)	3500 (± 420)	3500 (± 420)	3500 (± 420)	0
Exclusive breastfeeding^2^ (weeks)	19 (± 9.4)	20 (± 9.5)	18 (± 9.4)	3
Exclusive breastfeeding^3^, *n* (%)	211 (87%)	38 (86%)	173 (87%)	3
Age at the CFT assessment (years)	17 (± 7.8)	7.2 (± 0.84)	19 (± 7.1)	0
FI score (points)	110 (± 14)	110 (± 12)	110 (± 15)	0
Time between UIE and CFT (years)	3.5 (± 4.0)	0.71 (± 1.2)	4.1 (± 4.2)	0
UIE (μg/d)	94 (± 52)	59 (± 27)	100 (± 53)	0
UIE > EAR, *n* (%)	160 (66%)	18 (41%)	142 (72%)	0
Paternal higher education, *n* (%)	177 (73%)	39 (89%)	138 (70%)	3
Maternal higher education, *n* (%)	183 (76%)	43 (98%)	140 (71%)	3

^1^Data are shown as mean (± SD) or *n* (%)

^2^Exclusive breastfeeding duration (weeks) was defined as the number of weeks of both exclusive (breast milk only with no other food or drink) and predominant (breast milk in combination with water or water-based drinks) breastfeeding

^3^Participants which were fully breastfeeding (*i.e.* ≥ 3 weeks)

Abbreviations: *CFT* culture fair intelligence test; *EAR* recommended daily allowance; *SD* standard deviation; *UIE* urinary iodine excretion

**Fig. 2:**
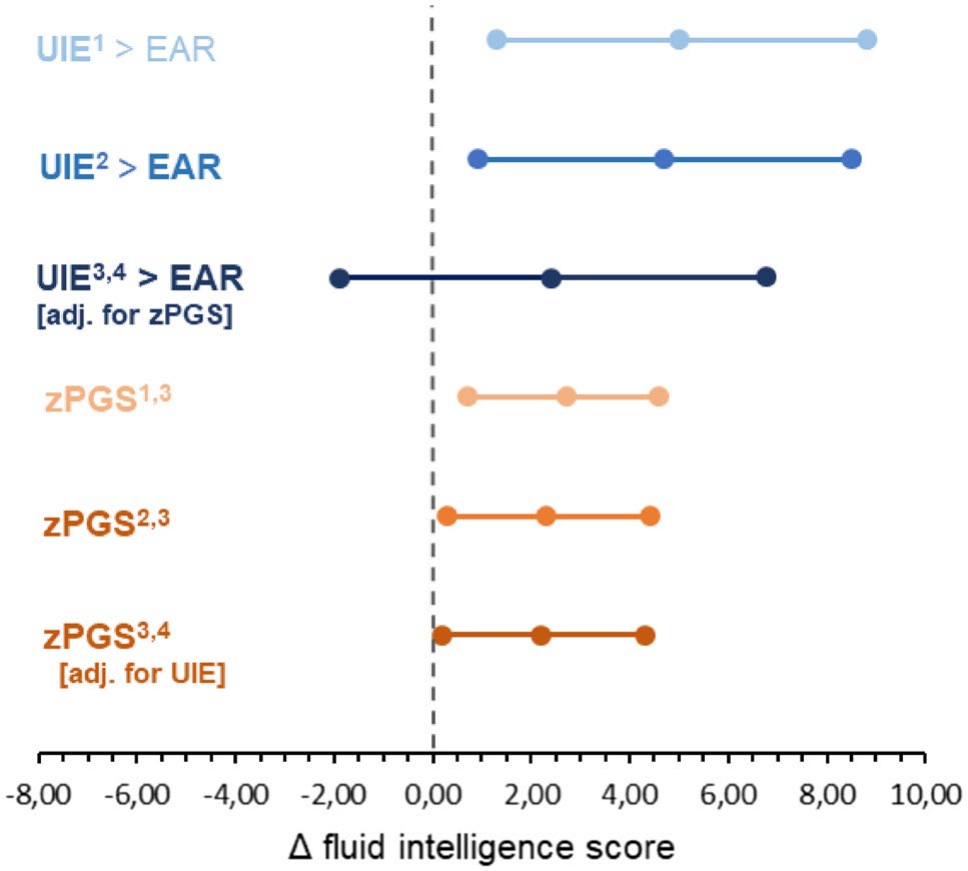
Associations between the FI score, UIE (blue), and PGS for general cognitive function (orange) in the DONALD study. ^1^Estimate (95%CI) obtained from the crude linear regression models. ^2^Estimate (95%CI) obtained from the linear regression models adjusted for age, birthweight, pregnancy duration and exclusive breastfeeding), time between the measurements, CFT version (CFT-1R vs. CFT-20-R) and parental higher education. ^3^Linear regression models including the PGS were additionally adjusted for the first five principal components. ^4^Estimate (95% CI) obtained from the linear regression models mutually including UIE and PGS adjusted for age, birthweight, pregnancy duration and exclusive breastfeeding), time in-between the measurements, CFT version (CFT-1R vs. CFT-20-R) and parental higher education. Abbreviations: *CFT* fair cultural intelligence test; *CI* confidence interval; *EAR* estimated average requirement; *FI* fluid intelligence; *PGS* polygenic score; *UIE* urinary iodine excretion above the EAR

An association between UIE and FI was observed in the univariable model. Specifically, participants with UIE ≥ EAR had five points higher FI than those with UIE < EAR (95% CI 1.3–8.8; *P* = 0.01). When age, birth factors, time between the measurements, CFT version and parental education were considered covariates, the association was attenuated to an average increase of 4.7 in FI for participants with UIE ≥ EAR (95% CI 0.9–8.5; *P* = 0.02). Likewise, the PGS was positively associated with FI. In the basic model, participants with higher PGS had an almost three points higher FI than participants with lower PGS (*β* = 2.7; 95% CI 0.7– 4.6; *P* = 0.01). The association was attenuated in the multiple regression model when further covariates were considered (*β* = 2.3; 95% CI 0.3– 4.4; *P* = 0.03). Then, we examined whether the association between FI and UIE, or FI and PGS changed substantially when the model was mutually adjusted for UIE and PGS. This showed that the association between UIE and FI was further diminished when the PGS was included as a covariate. Participants with UIE ≥ EAR had a higher FI score than those with UIE < EAR, although this association was no longer significant (*β* = 2.4; 95% CI – 1.9 to 6.8; *P* = 0.27). When the model was further adjusted for UIE, the association between the PGS and FI was only slightly attenuated. Participants with a higher PGS had a significantly 2.2 higher FI score than those with a lower PGS (*β* = 2.2; 95% CI 0.2–4.3; *P* = 0.04) (Fig. 2). No evidence of an interaction between UIE and PGS was observed (β =  − 0.7; 95% CI −5.3 to 3.9; *P* = 0.76).

**Fig. 3:**
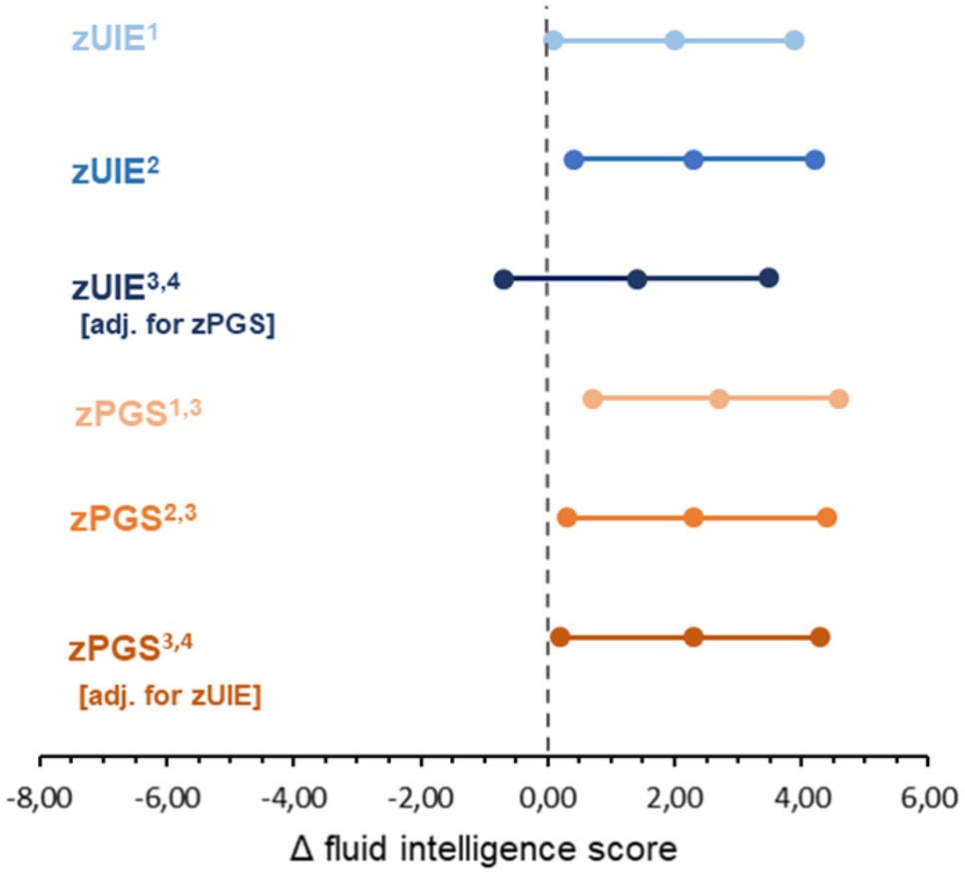
Sensitivity analysis: change in the FI score per 1 SD increase in either UIE (blue) or PGS for general cognitive function (orange) in the DONALD study. ^1^Estimate (95% CI) obtained from the crude linear regression models. ^2^Estimate (95% CI) obtained from the linear regression models adjusted for age, birthweight, pregnancy duration and exclusive breastfeeding), time in-between the measurements, CFT version (CFT-1R vs. CFT-20-R) and parental higher education. ^3^Linear regression models including the PGS were additionally adjusted for the first five principal components. ^4^Estimate (95% CI) obtained from the linear regression models mutually including UIE and PGS adjusted for age, birthweight, pregnancy duration and exclusive breastfeeding), time in-between the measurements, CFT version (CFT-1R vs. CFT-20-R) and parental higher education. Abbreviations: *CFT* fair cultural intelligence test; *CI* confidence interval; *EAR* estimated average requirement; *FI* fluid intelligence; *PGS* polygenic score; *UIE* urinary iodine excretion (z-score)

In the sensitivity analysis, where we included information from up to the latest three urine samples, the association of the mean UIE with FI was comparable, yet slightly attenuated, than the main analysis. Participants with UIE above the EAR had on average a four point higher score in the FI test than those with a UIE below the EAR in the univariable model (*β* = 4.1; 95% CI − 1.5 to 9.6; *P* = 0.15). The results remained comparable in the multiple regression model (*β* = 3.7; 95% CI − 1.8 to 9.3; *P* = 0.19). In line with the main analysis, no evidence of an interaction between UIE and PGS was observed (*β* =  − 1.3; 95% CI − 7.8 to 5.2; *P* = 0.70). Moreover, we observed comparable results when we used an age- and sex-standardised UIE (Fig. 3). A positive association was found between UIE and FI. Compared with participants having a mean UIE, those having UIE 1 SD higher had a 2.0 points higher FI score (95% CI 0.1–3.9; *P* = 0.04). The association was comparable in the multivariable model (*β* = 2.3; 95% CI 0.40– 4.20; *P* = 0.02). The association between zUIE and FI was further diminished when the PGS was included as a covariate (*β* = 1.40; 95% CI − 0.7 to 3.50; *P* = 0.18), whereas the association between the PGS and FI was virtually unchanged in this mutually adjusted model (*β* = 2.3; 95% CI 0.2–4.3; *P* = 0.03) (Fig. 3). Similarly, no evidence of interaction was found between UIE and PGS (*β* = 0.1; 95% CI − 1.9 to 2.1; *P* = 0.93).

## Discussion

Among children and adolescents from the German DONALD study, we observed that compared with children having UIE below the reference level, those having UIE above the EAR was associated with a five-point higher FI score. Given that we used up to three urinary iodine measurements, but not the habitual iodine intake throughout childhood, the urinary iodine may only reflect a proxy iodine intake given the time point during childhood and adolescence. In addition, the PGS was also positively associated with the FI score, as previously reported in adults [4]. Moreover, we investigated whether the magnitude of the association between the PGS and FI was comparable using a *z*-score-transformed age- and sex-standardised UIE. This analysis showed that the association between UIE and FI was comparable in magnitude to the association between PGS and FI. To examine the independence of these associations, we included both UIE and PGS in the same model and observed that the association of the PGS with FI was larger than those of the zUIE. Moreover, the association between zUIE and FI was no longer significant, which indicates that the association between UIE is particularly explained by the individual genetic disposition. However, our study did not suggest a modification of the association between UIE and FI by the PGS.

Despite wide investigations of the prenatal effects of severe iodine deficiency during the critical phases of brain development and the consequences of milder iodine deficiency on mental abilities [26], less is known about the postnatal effects of potential iodine deficiency on cognitive function. However, our results that participants with UIE > EAR have a higher FI are comparable with those of previous studies. A recent meta-analysis including 19 studies showed that compared with iodine-replete children, iodine-deficient children aged < 5 years had a lower IQ of 6.9–10.2 points [8]. Similar findings were observed in a meta-analysis including 37 studies (*n* = 12,291), which compared the effects of iodine on the intellectual development of children (aged ≤ 16 years) living in communities characterised as naturally iodine sufficient with children living in severely iodine-deficient areas or children in iodine-deficient areas born before and after the introduction of iodine supplementation. The authors observed that children exposed to severe iodine deficiency displayed a 12.5-point lower IQ. Moreover, compared with children whose mothers were persistently exposed to iodine deficiency, children receiving iodine supplementation had 8.7 points higher IQ than those receiving iodine supplementation during pregnancy [27]. While most of the aforementioned studies have investigated the effect of iodine deficiency on the critical phases of brain development, i.e. in the uterus and during the first life years, this study shows the importance of adequate iodine intake beyond this time frame. Thus, the observed positive association between UIE and FI during childhood throughout young adulthood adds to the existing body evidence on the importance of iodine intake for cognitive performance.

To investigate whether the individual genetic disposition may modify the association between iodine intake and FI, we calculated a PGS based on the reported genetic variants that were shown to be associated with general cognitive function in GWAS studies among up to 300,400 adults [4]. To our knowledge, these variants have not been used earlier to study the association with FI in children. In this study, we observed a similar positive association in children and adolescents as described previously in adults [4]. Notably, no evidence of an interaction between UIE and PGS was observed. Thus, no evidence shows that the genetic background of our study participants influenced the association between UIE and FI in our main analysis, when we considered the latest UIE, or in the sensitivity analysis, when a proxy of habitual UIE was considered by pooling up to three measurements of UIE. Although the association of UIE and the individual genetic background was comparable in magnitude (*β*: zUIE = 2.3 vs. PGS = 2.4) when considered individually, the sensitivity analysis revealed that individual genetic deposition appeared to attenuate the association between FI and UIE when mutually compared. The association between FI and the PGS was somewhat stronger than the association between FI and zUIE (*β*: zUIE = 1.4 vs. PGS = 2.3; Fig. 3). This might have been expected given that common genetic variants account for 22–46% of the phenotypic variation in childhood intelligence according to a previous GWAS [6]. Interestingly, the genetic influence on general cognitive ability increases with age. In a sample of 11,000 pairs of twins from four countries (*i.e*. UK, USA, Netherlands and Austria), heritability estimates for general cognitive ability increased significantly and linearly increased from 41% in childhood (age 9 years) to 55% in adolescence (age 12 years) and to 66% in young adulthood (age 17 years) [28]. In this sample from the DONALD study, no difference in FI scores between the younger (mean age, 7.2 ± 0.84 years) and older (mean age 19 ± 7.1 years) participants was observed. However, we adjusted our analysis for age because environmental influences increasingly account for individual differences in behaviour as experiences accumulate during the course of life [28].

Approximately, 85% of the iodine consumed is excreted in the urine, which makes UIE a highly suitable surrogate marker for iodine intake; thus, it is the recommended measure to monitor the iodine status within populations [29]. Interestingly, the intake of milk, fish, egg and meat was reported to predict UIE in the DONALD study [30]. Likewise, the intake of milk, egg and seafood, but not meat and poultry, was found to be positively associated with an estimated 24-h UIE in pregnant women living in the southeast of the UK [31]. However, we have not investigated whether these food groups were associated with FI. A previous study including 866 Scottish participants with a mean age of 72 years observed that participants with low intake of iodine-rich food items (*i.e*. dairy and fish) had increased brain volume shrinkage during 3 years of follow-up [32]. Likewise, fish consumption has been associated with slower cognitive decline in participants aged ≥ 65 years [33] and better cognitive performance in 2031 participants aged 70–74 years [34]. Moreover, dietary patterns including the Mediterranean diet [35] and the MIND diet [36] are beneficial for the development of Alzheimer’s dementia. However, less is known about the association between dietary patterns and cognitive abilities among children and adolescents. A recent meta-analysis including 21 studies that investigated executive functioning among children or adolescents aged 6–18 years concluded that a healthy dietary pattern (i.e. rich in fruits, vegetables, whole grains and fish) was positively associated with executive functioning, whereas less-healthy snacks, sugar-sweetened beverages and red/processed meats were inversely associated with executive functioning [37]. Albeit, it is commonly postulated that the beneficial associations observed between fish intake and cognitive abilities is dedicated to the high amount of long-chain n–3 PUFAs in oily fish [38] and not because of fish being rich in iodine. Nonetheless, iodine supplementation is beneficial for cognitive function among schoolchildren with iodine deficiency [9, 10]. Among children aged 3 to ≤ 6 years from the DONALD study, intake of milk (42%) and salt (42%) contribute the most to the amount of iodine excreted via the kidney, whereas the contribution of the intake of meat (7%), eggs (7%) and saltwater fish (2%) was modest [18]. Moreover, the DONALD study showed that a lifestyle score, comprising diet, physical activity, sedentary behaviour and sleep duration in childhood and adolescence, was positively associated with subsequently measured FI scores in participants aged 8.5–32 years [39]. The diet component included the intake of fruits, vegetables, wholegrain, sugar-sweetened beverages, fish, red meat and sausages, yet have not been investigated separately in relation to FI. Therefore, future studies investigating whether dietary patterns explaining the variation in iodine intake may have an influence on cognitive performance, particularly in younger individuals, would be interesting, not at least because iodine intake is the lowest among adolescents and young adults according to the German National Survey conducted from 2005 to 2006 [40], and recent data indicate a decline in the iodine status of German children [16, 41].

### Strengths and limitations

This study has some limitations. Cognitive function is a multifactorial phenotype; thus, the multivariable regression models included various confounders, but some were not possible to be considered in the DONALD study. First, low birthweight (< 2500 g) is associated with lower cognitive function in children [42], adolescents [43] and adults [44]. However, birthweight, within-the-normal-birthweight range (> 2500 g), was positively associated with cognitive ability [45]. Moreover, longer gestation was found to be associated with beneficial cognitive development, even among term infants [46]. Therefore, we adjusted our analysis for birthweight and pregnancy duration. Second, maternal iodine supplementation was found to be associated with cognitive function [26]. Information on maternal iodine supplementation was available for very few study participants (*n* = 2). Third, in the sensitivity analysis, up to three measurements of urinary iodine were pooled to examine a proxy of for habitual iodine exposure over time. To capture a reliable estimate, the habitual intake data from 10 to 24-h urine samples would have been needed [47]. Fourth, the assessment of FI via the CFT-1 and CFT-20R in the DONALD study has not been validated. However, the positive association with the PGS, which is based on genetic variants previously associated with general cognitive function [4], indicates that the CFTs served as a measure to differentiate between participants with lower from those with higher cognitive function. Fifth, the construction of the PGS might not be considered fully independent given that from known genetic variants [4], only those SNPs with a threshold of *P*-values < 0.3, based on the crude association between FI and the PGS within the DONALD study, were included in the PGS. However, the general selection of genetic variants included in the PGS was based on the previously identified genetic variants (within a linkage disequilibrium block (*r*^2^ = 0.2, range 1000 kb) associated with general cognitive function in a recent GWAS [4]. Sixth, the primary research aim of the DONALD study is to investigate dietary intake, lifestyle and anthropometrics; thus, the sample size for add-on modules added to the study programme was limited. However, our power analysis showed that the sample sizes of 238 and 162 participants, respectively, were sufficient to observe differences of two points in the FI score with a power of 80% considering an α-level threshold of 0.05. Nonetheless, the sample size was too small to observe an interaction of reasonable effect size with 80% power. Finally, the DONALD study is not necessarily representative of the general population with respect to the socio-economic status [12].

In conclusion, our study confirmed that both iodine intake and genetic predisposition were associated with cognitive function in children, adolescents and early adults. No evidence shows an interaction between iodine intake and genetic predisposition on cognitive function. Further studies, particularly studies that assess cognitive abilities more distinctly, i.e. including further cognitive domains in addition to FI, are warranted. Moreover, to capture the subtle but widespread adverse effects on populations that may occur secondary to hypothyroidism in the form of moderate-to-severe iodine deficiency (including decreased educability, apathy and reduced work productivity), long-term follow-up investigations in young to middle-aged participants are needed. Finally, it would be interesting to investigate whether different food groups rich in iodine or dietary patterns explaining variations in individual iodine intake are associated with cognitive performance.

## Data Availability

Data described in the manuscript, code book and analytic code of this study will be made available upon request. DONALD study data are available upon reasonable request for research questions within the scope of the DONALD Study, which are consistent with the legal and ethical standard practices of the DONALD study. Request should be addressed at epi@uni-bonn.de.
